# Oxidative Damage in Sporadic Colorectal Cancer: Molecular Mapping of Base Excision Repair Glycosylases in Colorectal Cancer Patients

**DOI:** 10.3390/ijms21072473

**Published:** 2020-04-02

**Authors:** Pavel Vodicka, Marketa Urbanova, Pavol Makovicky, Kristyna Tomasova, Michal Kroupa, Rudolf Stetina, Alena Opattova, Klara Kostovcikova, Anna Siskova, Michaela Schneiderova, Veronika Vymetalkova, Ludmila Vodickova

**Affiliations:** 1 Department of Molecular Biology of Cancer, Institute of Experimental Medicine of the Czech Academy of Sciences, Videnska 1083, 142 20 Prague, Czech Republic; Tomasova.Kristyna@seznam.cz (K.T.); misa.kroupa@gmail.com (M.K.); alenaopattova@gmail.com (A.O.); anna.siskova21@gmail.com (A.S.); veronika.vymetalkova@iem.cas.cz (V.V.); ludovod@seznam.cz (L.V.); 2 Biomedical Centre, Faculty of Medicine in Pilsen, Charles University, Alej Svobody 1655, 323 00 Pilsen, Czech Republic; 3 Institute of Biology and Medical Genetics, First Faculty of Medicine, Charles University, Albertov 4, 128 00 Prague, Czech Republic; marketk@seznam.cz; 4 Department of Biology, Faculty of Education, J Selye University, Bratislavska 3322, 945 01 Komarno, Slovakia; 5 Department of Toxicology and Military Pharmacy, Faculty of Military Health Sciences, University of Defence, Trebesska 1575, 500 01 Hradec Kralove, Czech Republic; r.stetina@tiscali.cz; 6 Laboratory of Cellular and Molecular Immunology, Institute of Microbiology of the Czech Academy of Sciences, Videnska 1083, 142 20 Prague, Czech Republic; 7 Department of Surgery, University Hospital Kralovske Vinohrady in Prague, Third Medical Faculty, Charles University, Ruska 87, 100 00 Prague, Czech Republic; schneider77@seznam.cz

**Keywords:** oxidative DNA damage, DNA repair, base excision repair (BER)glycosylases, colorectal cancer

## Abstract

Oxidative stress with subsequent premutagenic oxidative DNA damage has been implicated in colorectal carcinogenesis. The repair of oxidative DNA damage is initiated by lesion-specific DNA glycosylases (hOGG1, NTH1, MUTYH). The direct evidence of the role of oxidative DNA damage and its repair is proven by hereditary syndromes (MUTYH-associated polyposis, NTHL1-associated tumor syndrome), where germline mutations cause loss-of-function in glycosylases of base excision repair, thus enabling the accumulation of oxidative DNA damage and leading to the adenoma-colorectal cancer transition. Unrepaired oxidative DNA damage often results in G:C>T:A mutations in tumor suppressor genes and proto-oncogenes and widespread occurrence of chromosomal copy-neutral loss of heterozygosity. However, the situation is more complicated in complex and heterogeneous disease, such as sporadic colorectal cancer. Here we summarized our current knowledge of the role of oxidative DNA damage and its repair on the onset, prognosis and treatment of sporadic colorectal cancer. Molecular and histological tumor heterogeneity was considered. Our study has also suggested an additional important source of oxidative DNA damage due to intestinal dysbiosis. The roles of base excision repair glycosylases (hOGG1, MUTYH) in tumor and adjacent mucosa tissues of colorectal cancer patients, particularly in the interplay with other factors (especially microenvironment), deserve further attention. Base excision repair characteristics determined in colorectal cancer tissues reflect, rather, a disease prognosis. Finally, we discuss the role of DNA repair in the treatment of colon cancer, since acquired or inherited defects in DNA repair pathways can be effectively used in therapy.

## 1. Introduction

Colorectal cancer (CRC) represents significant social and public health problems, particularly in developed countries worldwide. At the most recent overview, colon cancer accounted for 1,096,601 new cases and 551,269 deaths in 2018, whereas rectal cancer was less frequent (704,376 newly diagnosed cases with 310,394 patients who died) [1]. Particular dietary and lifestyle habits and age constitute major risk factors in sporadic CRC, as recently reviewed [2,3].

Sporadic (non-hereditary) CRC (70–75% of CRCs cases) occurs in people without genetic predisposition or family history of CRC [4]. CRC often develops in genetically susceptible individuals as a consequence of the co-inheritance of multiple low-risk variants. Whereas up to 35% of interindividual variability in CRC risk has been attributed to genetic factors, high-risk germline mutations in *APC*, *MMR*, *MUTYH (MYH), SMAD4, BMPR1A* and *STK11/LKB1* genes account for about 6% of all cases [5,6].

Colorectal carcinogenesis includes three major genetic and epigenetic pathways: chromosomal instability (CIN), CpG island methylator phenotype (CIMP) and microsatellite instability (MSI). MSI is driven by functional impairment of DNA mismatch repair (MMR) genes and it is characterized by alterations in the length of microsatellites [4,7,8]. CIN is hallmarked by changes in chromosomal copy numbers. CIMP, as a form of epigenetic modification, refers to hypermethylation at repetitive CpG dinucleotides (so-called CpG islands) in the promoter regions of tumor suppressor genes (such as *MLH1*, *MINT1*, *MINT2* and *MINT3*) that silences gene expression [4]. Ample and convincing evidence has been accumulated on the role of inflammation, lipid peroxidation, oxidative stress and metabolic dysfunction in CRC onset and development (reviewed in [3]). Several physiological and pathological processes, closely linked to CRC development in the human body (i.e., obesity, diabetes, inflammatory bowel diseases), stimulate the formation of reactive oxygen species (ROS) and subsequent DNA damage [9]. For instance, obesity increases inflammatory factors and adipokines (TNF, leptin, IL-1β and IL-6), subsequently promoting oxidative stress and suppressing the immune system. These alterations often end up in aberrant cell signaling, increased cell growth and angiogenesis [10,11]. Disturbances in DNA damage levels, antioxidant status and capacity for DNA repair result in the accumulation of mutations and genomic instability. The involvement of dietary factors in the etiology of CRC suggests that this disease may be preventable by prudent dietary adjustments, e.g., by antioxidant-rich food [12,13] and optimal selenium uptake [14].

In this study, we intended to summarize our current knowledge on the role of oxidative DNA damage and its repair on the onset and prognosis and treatment of sporadic CRC, taking into account tumor heterogeneity. We also addressed the roles of glycosylases (hOGG1, MUTYH) involved in the base excision repair (BER) of oxidative damage.

## 2. Colorectal Cancer and Oxidative DNA Damage

### 2.1. DNA Damage and Colorectal Cancer Pathogenesis

Chronic human inflammatory diseases, diabetes, aging and various malignant diseases, including CRC, are hallmarked by an increase in oxidative DNA damage [15]. Oxidative stress belongs to the ubiquitous events attacking biologic systems. It has been postulated that oxidative stress is responsible for steadily increasing oxidative damage burden from early adenoma to CRC progression [16,17]. In general, unrepaired DNA damage and subsequent disruption in DNA damage response (DDR) pathway have been recorded in many cancer types and are responsible for genomic instability, a pivotal feature of cancer [18]. Indeed, Pearl et al. [19] have documented complex functional impairment in DDR in several cancer types. Importance of DNA repair pathways (a constituent part of DDR) in maintaining genomic instability and cancer etiology is highlighted in familial cancers with known high-penetrance germline mutations in DNA repair genes: *BRCA1/BRCA2* in breast cancer, MMR and polymerase deficiency (*MLH1*, *MSH2*, *MSH6, PMS2 and POLE* genes) in CRC and ovarian cancers, deleterious mutations in *RAD51C* and *RAD51D* and *BRCA1* mutation in ovarian cancers [20,21,22,23,24,25,26].

### 2.2. Oxidative DNA Damage, Characteristics, Biologic Properties and Relevance

ROS are engaged in many redox-governing processes of the cells in order to maintain cellular homeostasis and they pose potential signaling molecules to control several physiological cellular functions (for review see [27]). Its overproduction results in oxidative stress, responsible for a bulk of oxidative damage in DNA. ROS comprise a group of highly reactive chemical ions and molecules that includes oxygen radicals, non-radicals and hydrogen peroxide [28]. They are produced either endogenously during normal aerobic cellular metabolism or exogenously by agents such as ionizing radiation, chemotherapeutic drugs and transition metals. Elevated levels of ROS or depressed antioxidant defense lead to the imbalance in cellular DNA damage formation. ROS attack biologic macromolecules, resulting in DNA base and sugar damage, apurinic or apyrimidinic sites, DNA–protein cross-links and strand breaks, all contributing to genomic instability [29,30,31]. Once ROS reach DNA, the oxidation of nucleophilic DNA bases and the ribose sugar ring leads to base loss and strand breaks. Guanine is the most prominent target, giving rise to 8-oxo-7,8-dihydro-2′deoxyguanosine (8-oxo-dG) and 2,6-diamino-4-hydroxy-5-formamidopyrimidine (FAPY). ROS also react with adenine (8-oxo-7,8-dihydro-2’deoxyadenosine, 2-hydroxyadenine)—and to a lesser extent with thymine and cytosine. Eight-oxo-dG is the most pro-mutagenic consequence of ROS, causing G ˃ T transversion [32,33] and is commonly measured either as the base in DNA or as the nucleoside 8-oxo-dG in urine [29]. Oxidative DNA damage triggers multiple pathways that include DNA repair, cell cycle arrest and apoptosis. Under physiological conditions, the steady-state level between DNA damage, antioxidant status; capacity for DNA repair is established and critical mutations in cancer-related genes are rather rare events.

### 2.3. The Repair of Oxidative DNA Damage

Altered DNA repair, comprising BER, nucleotide excision repair (NER), MMR, direct DNA repair, homologous recombination repair (HR) and non-homologous end-joining repair, acts as an important player involved in both cancer initiation and progression [34,35]. Moreover, modulations in DNA repair processes contribute to genetic heterogeneity and cancer evolution (genomic/chromosomal instabilities). Relevance of DNA repair and DDR in cancer onset, its progression and patients´ therapeutic response has recently been reviewed in 33 cancer types. Genetic changes (mutations, loss of heterozygosity) were observed in 33% of DNA repair and DDR genes highlighting the participation of these pathways in tumorigenesis [36].

BER pathway is the main mechanism involved in the removal and repair of oxidized DNA bases (for reviews see [37,38]). The repair of oxidative DNA damage (including premutagenic 8-oxo-dG) is initiated by lesion-specific DNA glycosylases, such as hOGG1 and MUTYH, which are the first enzymes in this pathway responsible for locating and removing DNA single damaged base. Eleven DNA glycosylases have been identified in human BER so far (Table 1) [39]. The redundancy in the substrate specificities of the glycosylases that recognize and remove oxidized DNA bases supports robust and efficient cell defense against oxidative stress. It has developed in organisms to protect the genome from the perpetual attacks of oxygen radicals both under pathologies and physiological conditions.

**Figure 1:**
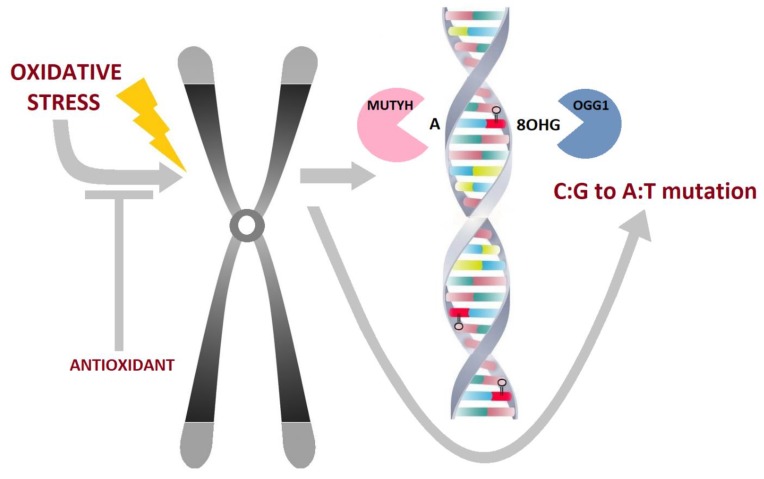
MUTYH and hOOG1 cooperate to prevent C:G to A:T transversion mutations under oxidative stress.

Human 8-oxo-dG DNA N-glycosylase 1 (hOGG1) removes 8-oxo-dG from the DNA and mutY DNA glycosylase (MUTYH, also termed MYH) excises misincorporated adenines opposite to 8-oxo-dG via replicative DNA polymerases α, δ and ε (reviewed in [55]). Both glycosylases suppress tumorigenesis by preventing mutagenic G:C > T:A transversions, as well as by inducing MUTYH-dependent cell death (reviewed by [56]). It should be noted that hOGG1 glycosylase acts as a counter partner of MUTYH (Figure 1). Both glycosylases stimulate consequent steps in BER (action of AP endonuclease I and Polymerase β) to complete the repair process by incision, gap-filling and ligation. However, other BER enzymes also participate to protect DNA against oxidative damage, such as NTH1, MTH1, NEIL1-3, XRCC1 and PARP-1 [57,58,59,60]).

Another DNA glycosylase involved in the excision of a wide spectrum of oxidized pyrimidines is endonuclease VIII-like 1 encoded by the *NEIL1* gene, that figures as a back-up for DNA glycosylase NTH1 [61]. *NTH1* germ-line variant D239Y (G > T substitution) has been found to induce genomic instability and cellular transformation in non-transformed human and mouse mammary epithelial cells [61,62].

One of the most important nucleases removing oxidized deoxynucleotide triphosphates (dNTPs) from the cellular pool is mutY homolog named human mutT homolog 1 (MTH1). Nucleotides in the pool are particularly vulnerable to oxidation and incorporated and unrepaired 8-oxo-dGTPs further contribute to G:C > T:A transversion. MTH1 preserves genomic integrity by preventing the incorporation of mutagenic purines into nuclear and/or mitochondrial DNA [63]. Depletion or inhibition of MTH1 also results in DNA strand breaks [64]. Additionally, MTH1 helps to protect telomeres, the essential structures at the end of chromosomes, from their oxidation and shortening and prevents the induction of genomic instability [65].

After the initial step of recognition and excision of oxidized bases by DNA glycosylases, DNA nicks occur that are sealed by DNA ligases. Several major types of DNA ligases (such as LIG1, LIG3, LIG4) have been discovered in human cells so far [66]. DNA nicks occurring during DNA replication or as the intermediate of BER are sealed by human DNA ligase I (LigI) or DNA ligase III (LigIII) along with XRCC1 DNA repair enzyme. High expression of *LigI* has been described in many human solid cancers [67]. The inhibition of LigI may therefore potentially block DNA replication and may also sensitize cancer cells to chemotherapeutic agents [68] leading to apoptosis [62,69].

Studies on laboratory animals have been intended to solve the question of whether oxidative damage occurs before or in the early stages of carcinogenesis or appears as a consequence of this process. The 8-oxo-dG has been detected in experimental animals treated with chemical carcinogens [70,71] or irradiated by X-rays [72] suggesting secondary oxidative stress that accompanies exposures to specific genotoxicants. In a comprehensive study of Olinski et al. the oxidized DNA bases were found in target organs of animals treated with various carcinogens (e.g., heavy metals) long before the tumor appeared [73]. Although the higher presence of 8-oxo-dG was not recorded in MUTYH knockout mice [74], simultaneous knocking out of both MUTYH and hOGG1 glycosylases resulted in a synergistic increase in G > T transversions [75]. All available evidence points to the enhanced oxidative DNA damage in tumors as a result of malignant transformation.

### 2.4. Oxidative DNA Damage, its Repair and Implications in Colorectal Carcinogenesis

#### 2.4.1. Hereditary Syndromes with Defects in Glycosylases Predisposing Colorectal Cancer

Hereditary syndromes with germline mutations in selected repair genes predispose to complete loss-of-function of BER proteins and thus facilitate the inactivation of oxidative DNA damage removal process which results in accumulation of oxidative DNA damage in the transition from early adenoma to CRC. These comprise MUTYH-associated polyposis (MAP) and NTHL1-associated tumor syndrome (NATS) [26,56]. Although these hereditary syndromes account for less than 2% of all CRC, they represent a substantial (80%) risk of CRC development in MAP patients and directly connect the oxidative damage and its repair with CRC development. MAP-associated CRC exhibits G:C>T:A mutations in tumor suppressor genes and proto-oncogenes and widespread occurrence of chromosomal copy-neutral loss of heterozygosity [76]. Recessively inherited mutations in the *NTHL1* gene cause a polyposis and CRC syndrome [77], about five times less frequent than MAP. This NATS syndrome is based on a unique, clearly distinct mutational signature, G:C > A:T transition at the non-CpG site [56]. The above findings raise the question of whether other BER glycosylases could be candidate genes for new, yet undiscovered polyposis syndromes.

#### 2.4.2. Sporadic Colorectal Cancer

In sporadic CRC, unrepaired 8-oxo-dG adducts induce mutations in proto-oncogenes, such as *KRAS* and tumor suppressor genes and, also, both *MUTYH* and *hOGG1* were found to be downregulated in neoplastic human colon tissues compared to adjacent tissues [78]. The concerted action of *MUTYH* and *hOGG1* is illustrated in Figure 1. This is in accordance with the suggestion that reduced BER capacity elevates susceptibility to oxidative DNA damage in various cancer types, including their invasiveness [79]. Unrepaired oxidative lesions, such as thymine glycol, can also stall the progression of a replicative fork and therefore contribute to genomic instability. Yet, another mechanism, based on DNA global demethylation mediated by BER in early colorectal tumorigenesis, has recently been postulated. The study of Furlan et al. found that MUTYH-associated polyposis adenomas exhibited strikingly pronounced hypomethylation than familial adenomatous and sporadic polyps. The authors concluded that DNA demethylation, together with specific KRAS/NRAS mutations, drives the early steps of oxidative damage related colorectal tumorigenesis [80]. In this context, we have not recorded any aberrant methylation in relevant DNA repair genes in tumor tissues from colorectal cancer patients [81]. Less information is currently available on MTH1 nuclease with regard to sporadic CRC. High expression of *MTH1* has been observed in many human malignancies [82,83], including CRC, where the enzyme bolsters survival of the malignant cells and acts therefore in procarcinogenic manner.

#### 2.4.3. Base Excision Repair Capacity in Sporadic Colorectal Cancer

Recent years witnessed attempts to determine individual DNA excision repair capacities (DRC) both in healthy subjects and CRC patients [38]. DRC emerges as one of the most complex biomarkers since it integrates a plethora of factors such as gene variants, gene expressions, the interplay of relevant glycosylases, the stability of gene products, the effect of inhibitors/stimulators, lifestyle and environmental factors. Such a biomarker is of key importance in the identification of cancer risk in sporadic malignancies that are substantially affected by gene-environment interactions including oxidative DNA damage [34,84]. Functional DNA repair assays also provide fundamental information about the capacity of the organism to cope with chronic exposure to numerous environmental and dietary genotoxicants. Oxidative DNA damage, corresponding base excision repair capacity (BER-DRC) and relevant gene variants were addressed in 182 CRC patients and 245 controls. Whereas the 326Ser/Cys OGG1 and the 324Gln/His as well as the 324His/His MUTYH genotypes were associated with an increased CRC risk, the decreased efficiency of DNA repair was correlated with the 399Gln/Gln XRCC1 and the 324His/His MUTYH genotypes occurrence in CRC patients. Due to the missing information for some enrolled CRC patients, the validation study is warranted [85]. In our laboratory, we have measured both NER- and BER-DRC in tumor tissues and adjacent bowel mucosa of 70 incident CRC patients. In another study, BER-DRC in tumor tissue did not differ from that in adjacent mucosa. There was a good correlation between BER-DRC in tumor tissue, adjacent mucosa and peripheral blood lymphocytes. BER-DRC was not influenced by sex and age and, most importantly, did not differ between the colon and rectal tumors. No statistical significance was found in BER-DRC based on the pathologic stage of the tumors and the expression levels of BER genes did not correlate with BER-DRC [86]. Current reports suggest that oxidative DNA damage may be removed from DNA also via NER [87,88] and the authors postulate that loss of NER function shares common features arising from BER defects, including cancer predisposition [89]. Despite we have recorded significantly higher NER DRC in tumor tissue from CRC patients in comparison to adjacent mucosa, we fail to ascribe this change to any specific DNA damage [86]. Most recently, we have addressed BER-DRC in paired samples of tumor tissue and non-malignant adjacent mucosa of 123 incident colon cancer patients concerning 5-fluorouracil (5FU) therapy. Interestingly, BER-DRC in non-malignant adjacent mucosa was positively associated with overall and relapse-free survival. Moreover, the overall survival (OS) of these patients was further improved in patients with a decreased BER-DRC in tumor tissue. The ratio of BER-DRC in tumor tissue and adjacent mucosa positively correlated with advanced tumor stage [79].

#### 2.4.4. Sporadic Colorectal Cancer and Gene Variants in Base Excision Repair

It was postulated that gene variants including single nucleotide polymorphisms (SNPs) in DNA repair genes may alter DNA repair function, including the function of BER glycosylases, modulate its capacity, induce genetic instability or deregulate cell growth and propagate cancer [90,91,92]. Earlier studies indicated that *hOGG1* Ser326Cys (rs1052133) SNP significantly affected BER-DRC [92]. However, the association of this SNP with CRC risk remains inconclusive: Recently the authors [93] reported on 727 CRC cases and 736 healthy controls from Taiwan significant association between *OGG1* Ser326Cys and *APE1* Asp148Glu (rs1130409) SNPs and an increased CRC risk. The authors concluded that *OGG1* and *APE1* SNPs are associated with stage- and sex-specific risk of CRC. An early study on 532 CRC cases and 532 matched controls [94] found an enhanced risk of CRC in smokers with *hOGG1* Ser326Cys polymorphism. Increased CRC risk was also reported in individuals simultaneously homozygous for the variant alleles of *APE1* Asn148Glu and *hOGG1* Ser326Cys [94]. A meta-analysis comprising 4174 cases and 6196 controls did not reveal any robust association between *hOGG1* Ser326Cys polymorphism and CRC, the authors however recommended further investigation [95]. Another rare nonsynonymous variant in *hOGG1* Gly308Glu (rs113561019) has been discussed in relation to the susceptibility to CRC. In a recent well-powered study, the authors found no evidence for the association of the above *hOGG1* polymorphism with CRC risk [96]. A meta-analysis by Zhang et al. evaluated 12 association studies and concluded that *hOGG1* Ser326Cys polymorphism does not associate with CRC risk [97]. However, its role in gene-gene interactions may not be ruled out.

In the study by Pardini et al. the authors investigated in 1098 sporadic CRC patients for prognostic effects of 3´-untranslated region polymorphisms (representing microRNA binding site) in BER genes. Interestingly, *NEIL2* rs6997097 polymorphism was associated with shorter survival and *NEIL3* rs1055678 polymorphism with CRC recurrence. The altered epigenetic regulation of the specialized glycosylases *NEIL2* and *NEIL3*, involved in the recognition of oxidized pyrimidines and transcription process, may further add to the understanding of the effect of oxidative DNA damage in colorectal carcinogenesis [98].

Single nucleotide polymorphism rs7689099 in the *NEIL3* gene was reported to modulate significantly survival of CRC patients. The NEIL3 encodes a DNA glycosylase involved in the first step of the BER pathway. Significantly elevated expression levels in tumors, compared to corresponding non-malignant tissues, were reported in 20 cancer sites, including CRC [99].

In a meta-analysis (comprising more than 8000 CRC cases and 6000 controls) Picelli et al. revisited the associations of rs3219484:G-A (MUTYH V22M) and rs3219489:G-C (MUTYH Q338H) polymorphisms with the risk of sporadic CRC. The associations with studied polymorphisms were, however, negative for all CRC as well as for colon and rectal cancer separately [100].

### 2.5. Colorectal Cancer, Oxidative Damage and Intestinal Microenvironment

Intestinal epithelial and immune cells are in permanent contact (interaction) with variable microbial inhabitants; these interactions result in modulations of numerous physiological and pathological processes [101]. Recent discoveries revealed that gut microbiome and CRC are tightly connected and during the disease, the composition and function of microbes can significantly differ [102,103]. Species unambiguously associated with colorectal carcinogenesis are reported in [104]. Intestinal bacteria induce proinflammatory and pro-carcinogenic pathways in colonic epithelium, produce genotoxins and ROS, promote host immune response disturbance and chronic inflammation and mediate the conversion of procarcinogens into carcinogens [105]. There are currently two hypotheses explaining the role of bacteria in colorectal carcinogenesis: (a) “driver-passenger” theory suggesting that certain intestinal bacteria (bacteria drivers) induce epithelial DNA damage and tumorigenesis; (b) dysbiotic microbial community with pro-carcinogenic characteristics via remodeling the whole microbiome initiates pro-inflammatory cascades and subsequent cellular transformation [106,107]. The former is illustrated by phylogenic group B2 of *Escherichia coli* identified as producers of colibactin (pks^+^ *E. coli*), a peptide-polyketide, which induces several DNA adducts including those with bulky character, ultimately leading to inter-strand crosslinks or double-strand breaks [108]. The latter relates to a generation of ROS resulting in oxidative DNA damage [109,110]. Additionally, ROS are often produced in high amounts by tumor cells and influence local microenvironment and immune response [109]. The tumor microenvironment is composed of myeloid (innate immunity) and lymphoid (adaptive immunity) lineages. Infiltrating immune cells can function to control tumor growth or to help create an immunosuppressive environment in which the tumor can thrive. Recent understanding points to the fact that carcinogenesis shows many similarities to chronic inflammatory processes [111]. For instance, ulcerative colitis and CRC in relation to ROS were studied in mice deficient for epithelial-to-mesenchymal transition factor ZEB1 and DNA glycosylase MPG. Zeb1-deficient mice were partially protected from experimental colitis and, in a model of inflammatory CRC, they developed fewer tumors and exhibited lower levels of DNA damage (8-oxo-dG) and higher expression of *MPG* encoding DNA-3-methyladenine glycosylase [112]. The dynamic of microenvironment has been documented by different microbiota in relation to histology of adenoma/polyps. More strikingly, normalization of the microbiota has been recorded after colorectal cancer treatment. The intestinal microenvironment is further modulated by different bacterial strains due to the impacted generation of bacterial metabolites and toxins [113].

#### Oxidative Damage, Intestinal Microenvironment and CRC Prevention

Under physiological condition, a dynamic steady-state between ROS generation, antioxidant status, the formation of DNA damage and capacity for DNA repair, is constantly influenced by diet. However, the mechanisms by which nutritional components influence colorectal carcinogenesis are not yet clear. Since the first line of defense against ROS is the cellular antioxidant system, supplementation of volunteers’ diets with antioxidants or antioxidant-rich foods has led in many trials to decreases in the level of endogenous oxidation of DNA bases [12,114] and increased resistance to oxidative damage ex vivo [115]. For example, fruit consumption has been suggested to increase DNA repair capacity and decrease DNA damage, likely due to antioxidants and bioactive compounds in fruits [13]. Soymilk in a form of yogurt also exerts substantial antioxidant potential [116]. Experimental and observational evidence indicate that sub-optimal dietary intakes of selenium may contribute to increased risk for several tumors including CRC, through oxidative and inflammatory response selenoproteins which require selenium for their biosynthesis [14,117]. Moreover, the diet may also significantly modulate BER and NER capacities, for instance, fruit- and vegetable-rich diet stimulates the repair of oxidative DNA damage [9]. It has been documented that diet substantially affects the composition of gut microbiota [118,119,120]. Various diets not only alter the abundances of several bacterial strains but also change the metabolic profile of whole microbiota, e.g., increase in the biotransformation of pro-carcinogenic polycyclic aromatic hydrocarbons, formed during meat processing [121]. Microbiota respond differently to dietary components: for instance, protein-rich diet correlates with *Bacteroides*, diet rich in fiber correlates with *Prevotella* [122] and consumption of dietary fiber is associated with increased fermentation of indigestible plant polysaccharides [122]. Therefore, certain dietary intervention impacts the gut microbiota and could promote changes that are either harmful or beneficial to health, and thus could influence cancer incidence by limiting the development/relapse of the disease.

## 3. Possible Utilization of Oxidative DNA Damage in Colorectal Cancer Therapy

The therapy of CRC has comprehensively been reviewed [38,123]. Despite all the efforts in CRC therapy over the years, 5-year survival remains unsatisfactory. The prognosis for CRC patients decreases with increasing TNM staging, the five-years survival rate is up to 90% for stage I, but only less than 15% for stage IV [99]. CRC treatment usually involves complete primary tumor resection and appropriate chemotherapy, which often causes severe adverse effects [124]. There are considerable interindividual differences among CRC patients in the response to the therapy, probably due to inherited genetic susceptibility, acquired resistance of tumor cells and the role of DNA damage/DNA repair in chemotherapy [125,126].

Cancer cells are due to their hypermetabolic activity highly sensitive to the oxidative balance and have a high antioxidant capacity [127]. Therefore, anticancer therapy targeting antioxidant defense of cancer cells or generating ROS represents an interesting approach in CRC treatment strategy. Oxaliplatin in combination with piperlongumine, a natural product constituent of the fruit of the Long pepper (*Piper longum*), has been shown to act synergically and induce CRC cell apoptosis via mitochondrial dysfunction and endoplasmic reticulum stress [128]. The mode of piperlongumine action in experimental colon cancer has recently been updated [129]. Our results showed that co-treatment of CRC cells with 5FU and *Ganoderma Lucidum* induces oxidative DNA damage in CRC cell lines. Moreover, the non-malignant cells were protected against oxidative DNA damage [130]. Co-treatment with paclitaxel and lentinan exerts synergistic apoptotic effects in A549 cells through inducing ROS production [131]. The anticancer effects of natural compounds and their tentative modes of action have recently been reviewed [118].

Efficient DNA repair often confers poor response to chemotherapy and worse prognosis [79]. DNA-alkylating agents used in CRC therapy—such as temozolomide (treatment of metastatic CRC [132])—induce DNA lesions repaired by BER [132]. Suppression of the BER pathway by inhibiting polymerase β activity may also represent a tool for improving therapy response [133].

More interestingly, *MTH1* overexpression has been previously associated with distinct cancer stages and survival of the cancer patients [134,135]. The important role of MTH1 during malignant transformation and an increasing number of articles on this topic resulted in the discovery of potent and selective MTH1 inhibitor [136], currently in phase I clinical testing. Unfortunately, selective inhibition of MTH1 in lung cancer cells showed increased oxidative DNA damage which indicates that MTH1 inhibition will likely not be utilized as an across-the-board therapeutic strategy [137].

## 4. Discussion

There are currently serious disputes and unresolved questions regarding the etiology of sporadic CRC: is genomic and/or chromosomal instability a cause or consequence of tumorigenesis, is the alteration of microbiota preceding CRC onset or it appears as a consequence of it. Another enigmatic aspect is whether high levels of the oxidative damage found in CRC tumors are associated with early stages of carcinogenesis or rather with its consequences. The outcomes from laboratory animals addressing oxidative bases in carcinogenesis are rather inconclusive, so is the issue of oxidative DNA damage in target/surrogate tissues. The bulk of studies so far reported DNA damage and DNA repair in CRC considered as a single entity. However, recent investigations highlighted the differences in embryogenesis, etiology, anatomy, genetics and treatment response between the colon and rectal cancers with additional impact on prognosis for patients and different treatment strategies [138,139,140].

Recent studies on hereditary polyposis syndromes (MAP polyposis, NTH1 polyposis; [56,141]) leading to CRC provided unambiguous evidence on the role of oxidative DNA damage and lack of function BER glycosylases in CRC etiopathogenesis. However, some mechanistic aspects have not fully been clarified yet, e.g., *NEIL1*, *NEIL2* and *NEIL3* triple-knockout mice were not prone to cancer and do not have increased mutational frequency produced by defective BER [142]. Further, recent studies disclosed the role of MTH1, which prevents incorporation of oxidized purines into DNA, in malignant transformation [143]. However, high expression of *MTH1* has been observed in many human malignancies [82,83], including CRC, where the enzyme bolster survival of the malignant cells. These seeming discrepancies may be explainable by the versatile role of oxidative DNA damage in carcinogenesis: its higher extent may trigger a malignant transformation in very early stages, whereas in developed tumors, its lower level (or efficient BER) may give additional survival/growth advantage to cancer cells [144]. Studies aimed at the comparison of BER capacity in tumor tissues and adjacent mucosa from sporadic CRC patients did not show major differences in BER between these tissues [86]. However, the recent study by Vodenkova et al. pointed to the importance of the ratio between BER capacity in tumor tissue and adjacent mucosa among CRC patients. Low BER in tumor and higher BER capacity in adjacent mucosa conferred to significantly longer survival and vice versa. Additionally, the ratio of BER capacity in tumor tissue over BER capacity in mucosa correlated positively with the advanced tumor stage [79]. Although relatively well-characterized at present, oxidative DNA damage and its repair warrants further investigation in complex diseases. Regarding sporadic CRC, last years witnessed an advent of additional questions closely related to oxidative DNA damage and its repair. One of those concerns the target and surrogate tissues, in which this damage occurs. Peripheral blood lymphocytes have been taken for long merely as a surrogate in biomonitoring studies. However, these cells represent an important player in the immune system, the last gatekeeper of cancer progression. Further, the generation of oxidative DNA damage is significantly affected by the intestinal microenvironment (microbiota) and this microbiota have been shown to determine immune response. On the other hand, tumor cells produce in high amounts ROS, with subsequent effects on the above systems [109].

Several association studies addressed the role of low penetrance BER gene variants in sporadic CRC with often controversial results. The recent whole genome association studies failed to identify these variants in large CRC patient cohorts [5,145]. Despite it was shown that h*OGG1* 326Cys SNP significantly affected BER DRC, the overall effect is rather minor, and these gene variants may rather find their relevance in interactions [146,147].

In our recent review, we have summarized the role of DNA repair in the treatment of colon cancer [148]. Acquired or inherited defects in DNA repair pathways can be effectively utilized in the therapy; for instance, CRC patients bearing deficiency in *RAD51C* or *CHEK2* genes, belonging to HR pathway, benefited from the treatment with olaparib [149]. This drug inhibits PARP1, a protein implicated in BER, which is often over-expressed in various types of cancers. Tumors with dysfunctional HR may be dependent on PARP1-facilitated DNA repair and are sensitive for its inhibition [150]. However, olaparib-based therapies were tested mainly for breast, ovarian, pancreatic and prostate cancer cases with a mutation in high-penetrance genes BRCA1 and BRCA2 [151]. As evidenced in pre-clinical studies, hLigI may represent an additional attractive target for inhibition in rapidly dividing malignant cells [68]. Despite some promising results, not a single inhibitor is currently applied in clinical practice [152].

Oxidative DNA damage and activities of glycosylases and ligases involved in its repair still await their full application in therapeutic strategies of CRC.

## 5. Conclusions

The pivotal role of ROS in both health and disease has been recognized. Arising oxidative DNA damage represents an important factor in the etiopathogenesis of CRC and further effort should be dedicated to its monitoring. Similarly, it may also represent a significant marker of prognosis and its level may contribute to treatment outcome.

With increasing knowledge on the role of microenvironment in colorectal carcinogenesis, proper attention should be given to the dynamic of DNA damage formation (and oxidative DNA damage in particular) and its repair.

However, binary roles of ROS and emerging oxidative DNA damage may be utilized in cancer therapy by exploiting combinations of conventional chemotherapeutics with substances leading to oxidative DNA damage in CRC cells. Our recent study suggests that higher capacity of BER in adjacent mucosa and lower in tumor cells accompanied longer survival and good prognosis (and vice versa) of CRC patients. Scarce data are available on the extent of oxidative DNA damage in colorectal tumor tissues and adjacent mucosa.

## Figures and Tables

**Table 1:** List of human DNA glycosylases and their function.

Glycosylase Name	Gene	Enzyme Commission Number	Biologic Function	Reference
Adenine DNA glycosylase	*MUTYH*	3.2.2.31	MUTYH is a monofunctional DNA glycosylase which, after the replication, removes adenines mispaired with 8-oxo-dG.	Koger et al., 2019 [40]
N-glycosylase/DNA lyase	*OGG1*	4.2.99.18	OGG1 acts in cooperation with MUTYH. It is a major glycosylase for the removal of 8-oxo-dG. It possesses also an intrinsic AP lyase activity at abasic sites.	Wang et al., 2018 [41]
DNA-3-methyladenine glycosylase	*MPG*	3.2.2.21	MPG removes a variety of alkylated (3-methyladenine, 7-methylguanine) and deaminated (hypoxanthine) purines. It also recognizes and removes secondary oxidative lesions such as 1,N6-ethenoadenine.	Leitner-Dagan et al., 2012 [42]
Methyl-CpG-binding domain protein 4	*MBD4*	3.2.2.-	MBD4 preferentially binds to CpG sites and guards DNA against deamination of cytosine to uracil or 5-methylcytosine to thymine.	Sjolund et al., 2013 [43]
Single-strand selective monofunctional uracil DNA glycosylase	*SMUG1*	3.2.2.-	SMUG1 belongs to the uracil DNA glycosylase superfamily. It is a back-up uracil DNA glycosylase removing a wide variety of oxidized pyrimidines such as 5-hydroxyuracil, 5-hydroxymethyluracil, 5-formyluracil and 5-carboxyuracil In addition to that, SMUG1 has also an activity towards 5-fluorouracil, a commonly used chemotherapeutic agent to treat CRC.	Nagaria et al., 2013 [44], Alexeeva et al., 2019 [45]
Endonuclease III-like protein 1	*NTH1*	4.2.99.18	NTH1 cleaves a broad range of lesions such as thymine glycol, 5-hydroxyuracil, 5-formyluracil, 5-hydroxycytosine, 5-hydroxy-6-hydrothymine, 5,6-dihydroxycytosine, 5,6-dihydrouracil and formamidopyrimidine.	Shinmura et al., 2019 [46]
Endonuclease VIII-like 1	*NEIL1*	4.2.99.18	NEIL1 acts at the replication fork and it is implicated in direct removal of the 5-carboxylcytosine. Further, it stimulates TDG-mediated excision of 5-formylcytosine and 5-carboxylcytosine.	Slyvka et al., 2017 [47]
Endonuclease VIII-like 2	*NEIL2*	4.2.99.18	NEIL2 takes part in the transcription-coupled BER. It excises 8-oxoguanine, thymine glycol, formamidopyrimidine lesions and oxidative products of cytosine, particularly 5-hydroxyuracil and 5-hydroxycytosine.	Sarker et al., 2014 [48], Han et al., 2019 [49], Minko et al., 2019 [50]
Endonuclease VIII-like 3	*NEIL3*	4.2.99.18	NEIL3 acts preferentially on ssDNA. It removes spiroiminodihydantoin and guanidinohydantoin, further oxidation products of 8-oxo-7,8-dihydroguanine. It is also implicated in the repair of formamidopyrimidine DNA adducts.	Massaad et al., 2016 [51], Minko et al., 2019 [50]
G/T mismatch-specific thymine DNA glycosylase	TDG	3.2.2.29	TDG recognizes U-G or T-G mismatches caused by the deamination of the cytosine or 5-methylcytosine. Therefore, it prevents the formation of a C→T mutation. Further, it excises oxidized products of the 5-methylcytosine and 5-hydroxymethylcytosine, such as the 5-formylcytosine and 5-carboxycytosine.	Da et al. 2018 [52], Fu et al., 2019 [53]
Uracil-DNA glycosylase	UNG	3.2.2.27	UNG hydrolyzes uracil from both ss and dsDNA, leaving an apyrimidinic site. Such lesions can arise due to deamination of cytosine or due to misincorporation of dUMPs during replication or repair.	Weiser et al., 2018 [54]

## Abbreviations

5FU	5-fluorouracil
8-oxo-dG	8-oxo-7,8-dihydro-2´deoxyguanosine
BER	base excision repair
CIMP	CpG island methylator phenotype
CIN	chromosomal instability
CRC	colorectal cancer
DDR	DNA damage response
dNTPs	deoxynucleotide triphosphates
DRC	DNA excision repair capacity
EFS	event-free survival
FAPY	2,6-diamino-4-hydroxy-5-formamidopyrimidine
HR	homologous recombination repair
LigI	human DNA ligase I
LigIII	human DNA ligase III
LOH	loss of heterozygosity
hOOG1	human 8-oxo-dG DNA N-glycosylase 1
MAP	MUTYH-associated polyposis
MMR	mismatch repair
MSI	microsatellite instability
MSS	microsatellite stable
MTH1	human mutT homolog 1
MUTYH, MYH	mutY DNA glycosylase
NATS	NTHL1-associated tumor syndrome
NER	nucleotide excision repair
OS	overall survival
ROS	reactive oxygen species
SNP	single nucleotide polymorphism
